# Lateral Operation after Lithotrity in a Child

**Published:** 1842-07-16

**Authors:** 


*Lateral Operation after Lithotrity in a Child.*—At the Royal Academy of Medicine, session of April 5th, 1842, M. Guersant, Jun., presented a child, 10 years of age, on whom he had performed the operation of lithotomy. The patient had twice undergone the operation of lithotrity, at the ages of four and eight years, but symptoms of stone returned after each apparent cure. The child was at last admitted in the hospital, where he performed the bilateral operation, and removed ninety-one fragments of calculus; to effect this he was compelled to introduce the forceps thirty-two times, and the scoop five times; but although the operation was so tedious the child recovered perfectly.

